# Cloning and spatiotemporal expression of *Xenopus laevis Apolipoprotein CI*

**DOI:** 10.1371/journal.pone.0191470

**Published:** 2018-01-18

**Authors:** Jyotsna Sridharan, Tomomi Haremaki, Daniel C. Weinstein

**Affiliations:** Biology Department, Queens College of the City University of New York, Flushing, New York, United States of America; University of Colorado Boulder, UNITED STATES

## Abstract

Apolipoprotein CI (ApoCI) belongs to the Apolipoprotein superfamily, members of which are involved in lipid transport, uptake and homeostasis. Excessive ApoCI has been implicated in atherosclerosis and Alzheimer’s disease in humans. In this study we report the isolation of *Xenopus laevis apoCI* and describe the expression pattern of this gene during early development, using reverse transcription polymerase chain reaction and whole mount *in situ* hybridization. *Xenopus apoCI* is enriched in the dorsal ectoderm during gastrulation, and is subsequently expressed in sensory placodes, neural tube and cranial neural crest. These data suggest as yet uncharacterized roles for ApoCI during early vertebrate embryogenesis.

## Introduction

Apolipoproteins are protein constituents that bind to lipids via their amphipathic α-helical domains, forming lipoproteins of varying size and density [1]. They function in lipid metabolism by facilitating the transport, redistribution, and uptake of lipids between different tissues and by acting as cofactors for enzymes involved in lipid metabolism. The major apolipoproteins include apoAI, apoAII, apoAIV, apoB, apoCI, apoCII, apoCIII and apoE.

Human APOCI, at 6.6KDa, is the smallest member of the APOC subfamily; it is synthesized as a mature 57-residue peptide after cleavage of an N-terminal 26 amino acid signal peptide [2]. In circulation, APOCI is found as a surface component of High Density Lipoproteins (HDLs), Very Low Density Lipoproteins (VLDLs), and chylomicrons, and is exchanged between these forms during lipid metabolism [3].

APOCI is best known for its role in lipoprotein metabolism, primarily affecting intravascular turnover of triglyceride-rich lipoproteins (TRLs) that transport endogenous fat from the liver (VLDLs) and dietary fat from the intestine (chylomicrons). ApoCI inhibits hepatic remnant clearance by preventing ApoE-mediated binding and uptake of VLDLs by LDL receptor Related Protein (LRP), VLDL receptor (VLDLr) and LDL receptor (LDLr), and by inhibition of lipoprotein lipase activity [4–7]. Consistently, both endogenous mouse ApoCI expression in an *ApoE* deficient background and overexpression of human *APOCI* in either a wild type or an *ApoE* deficient background can lead to hypertriglyceridemia and atherosclerosis in mice [5, 8, 9]. Moreover, in humans, high APOC1 content of TRLs is considered a risk factor for early atherosclerosis [10].

Human APOCI is strongly expressed in the liver, and is also expressed in the lung, skin, spleen, adipose tissue, and brain [11]. In the brain, a polymorphism in the *APOCI* allele that leads to an increase in APOCI expression has been implicated as a risk factor in the pathogenesis of Alzheimer’s disease (AD) [12–14]; consistently, expression of human *APOCI* in mice can lead to learning and memory impairment, perhaps as a result of alterations in brain lipid metabolism or by interfering with ApoE binding to β-amyloid peptides [12, 13, 15, 16].

Despite our understanding of apolipoprotein function in lipid homeostasis and disease, the role of this protein family during early development has not been extensively addressed. Here, we describe the cloning and expression analysis of a*poCI* in embryos of the frog *Xenopus laevis;* our studies suggest important and perhaps conserved roles for ApoCI during gastrulation and subsequent patterning of the vertebrate ectoderm.

## Materials and methods

### All procedures were approved by the Queens College IACUC committee, protocol #160

#### 1. Description of procedures

The animal system used for these studies is the African clawed frog, *Xenopus laevis*. Adult female and male frogs (over 1 year old) were used to produce embryos for the proposed experiments. I maintain a colony of about 135 female and 35 male frogs; veterinary care is provided by the Queens College Vivarium Facility, under the directorship of Marie Birne. The colony is inspected and cared for on a daily basis. To generate embryos, female frogs were injected with 600–800 units of human chorionic gonadotropin (HCG). 12 hours after injection, female frogs were gently squeezed to express eggs from the cloaca. The females were allowed to rest for 3–4 months between each spawning. Eggs were fertilized in vitro; to obtain sperm, male frogs were anesthetized with 3-aminobenzoic acid ethyl ester. A midline incision was made and testes were removed. While under anesthetic the male was sacrificed by decapitation, double pithed, and disposed of according to the guidelines of the American Veterinary Medical Association. The testes are viable for approximately two weeks at 4°C; thus, testes from a single male were enough to fertilize eggs from about 20 females.

The research described here focuses on the study of early *Xenopus laevis* embryos, which were derived via in vitro fertilization and examined up to early tadpole stages (within approximately 48 hours after fertilization). To induce egg-laying, female frogs were primed by subcutaneous injection of 800uL recombinant human chorionic gonadotropin. 12 hours after injection, eggs were extruded by gently squeezing the females (this procedure is painless, and mimics the action of the male of the species during mating). Eggs were harvested in glass Petri dishes and fertilized by application of sperm from minced testes of *Xenopus* males—synchronous fertilization, required for these studies, can only be achieved via in vitro fertilization, necessitating the killing of males for testes removal. Males were euthanized prior to testes extraction; euthanasia was performed by decapitation following anesthesia, via tricaine methanesulfonate (TMS) immersion. For these studies, we sacrificed approximately 1 male frog every other week (sperm/testis culture can last up to 14 days at 4 degrees Celsius). Embryos were euthanized by immersion in an anesthetic overdose of TMS.

#### 2. Justification for use of animals

We utilized biochemical and molecular biological (“in vitro”) approaches in these studies; however, elucidation of the dynamic processes underlying early development also require an understanding of gene and cell regulation in both space and time, a resolution not generally provided by tissue culture experiments. We therefore must also study gene and protein function in the context of the intact, developing organism.

Embryos of the frog *Xenopus laevis* have a number of distinct advantages over other vertebrate developmental model systems. First, the source of biological material is abundant, since it is possible to generate a large number of embryos (thousands per day) that can grow in a simple buffered solution. Second, fertilization and embryonic growth in *Xenopus* is external, greatly facilitating the analysis of early developmental events. Third, early stage *Xenopus* embryos are large, and thus well-suited for microsurgical and microinjection techniques. Finally, and perhaps most important, a large body of experimental embryological data has been gathered from this model organism; in fact, much of our current understanding of early vertebrate embryology comes from the study of the amphibian embryo.

#### 3. Procedures to limit discomfort, distress, pain, and injury

Some of the embryological studies described here involve eggs and embryos at blastula and gastrula stages of development, prior to formation of the nervous system; thus, these embryos are not in discomfort, stress, or pain. For those experiments that required the use of older embryos, animals were anesthetized in a solution of 3-aminobenzoic acid ethyl ester prior to fixation. Generating eggs is not a painful process for the female: the gentle squeezing required to release the eggs actually mimics the arm movements of the male during natural mating. The males required for this study were anesthetized with 3-aminobenzoic acid ethyl ester prior to sacrifice. Minimizing discomfort and stress for the colony is a requirement for generating healthy embryos; our frog facility has state of the art equipment for regulating water quality, pH, aeration, light, and temperature, in order to maintain the colony under optimal conditions.

#### 4. Method of euthanasia

The only adult animals sacrificed for this research were approximately one male frog per week. Additionally, tadpole stage embryos were sacrificed for some of these studies. As described above, sacrifices were performed under complete anesthetic conditions so as to minimize pain, using methods consistent with the recommendations of the American Veterinary Medical Association (AVMA) Guidelines for the Euthanasia of Animals.

### Cloning and isolation of *Xenopus apoCI*

*Xenopus* Apolipoprotein CI was isolated as a gene upregulated in ectodermal cells expressing an Engrailed repressor-HNF3β fusion protein in stage 11 *Xenopus* embryos. Library construction and screening was performed using a modified protocol originally described in [17]. Full-length Apolipoprotein CI was subsequently cloned from a λZapII Stage 28 head library [18] using the following primers: 3-2U: 5’-GATACAAAGTGACTCATC; 3-2D: 5’-GGCTCACTGTTGTGCAAA.

### RNA preparation and RT-PCR

*Xenopus laevis* embryos were staged according to [19] and harvested at appropriate stages according to morphological criteria. Total RNA was prepared using RNA Bee RNA isolation reagent from Tel-Test Inc. RT-PCR was performed as described [20]. Primers used in this study are as follows:

ODC-U: 5’-AATGGATTTCAGAGACCA

ODC-D: 5’-CCAAGGCTAAAGTTGCAG

Chordin-U: 5’-CAGTCAGATGGAGCAGGATC

Chordin-D: 5’-AGTCCCATTGCCCGAGTTGC

3-2(*apoCI*)-U: 5’-GATACAAAGTGACTCATC

3-2(*apoCI*)-D: 5’-GGCTCACTGTTGTGCAAA

### *In situ* hybridization

Whole mount *in situ* hybridization was carried out using standard protocols [21]. For preparation of antisense *apoCI* RNA probe, pBluescript SK+ 3–2 plasmid vector was linearized with Not I and transcribed *in vitro* with T7 RNA polymerase. For double *in situ* hybridization, probes were synthesized using digoxygenin or fluorescein RNA labeling mix (Roche) and detected with corresponding antibodies (Roche). First antibody reactions were quenched using 0.1M glycine-HCl, pH2.2 [22]. Magenta phosphate (Sigma), BCIP (Roche) and BM Purple (Roche) were used for chromogenic reactions. Other probes used in this study were *Otx-A*, *Pax-6* and *Slug* [23, 24]. 40 μM vibratome sections were obtained by embedding 4% paraformaldehyde-fixed embryos in 20% type B Bovine Gelatin (Sigma).

## Results

**Fig 1:**
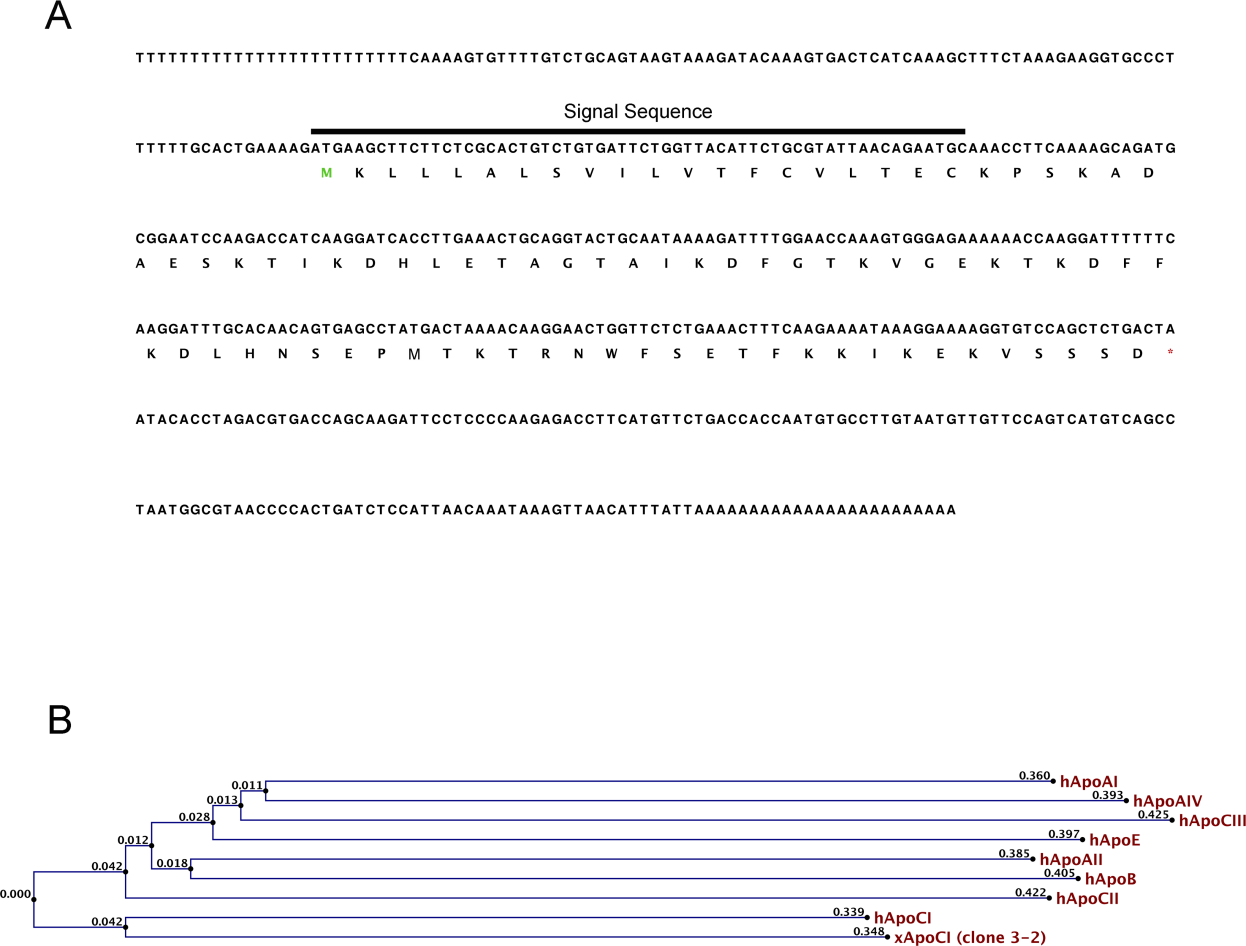
Sequence analysis of *Xenopus apoCI*. (A) Nucleotide sequence and deduced amino acid sequence of *Xenopus apoCI* (GenBank accession number: GU562893). Predicted signal sequence of *Xenopus* ApoCI spans residues 1–20. (B) Phylogenetic tree depicting evolutionary relationship of human Apolipoproteins AI, AII, AIV, B, CI, CII, CIII and E to *Xenopus Apolipoprotein CI*. The tree was constructed using the UPGMA distance matrix method from CLC Bio Workbench. Numbers at nodes indicate branch length.

Differential screening was used to identify transcripts enriched in dorsal tissues of gastrula stage *Xenopus laevis* embryos. One transcript isolated from this screen, clone 3–2, shares highest homology with members of the Apolipoprotein family from higher vertebrates. The predicted translation product of this cDNA (Fig 1A) is most closely related to human Apolipoprotein CI (hAPOCI; 38%). 3–2 shares significantly less sequence identity with human APOC2 (20%), APOC3 (17%) or other human apolipoproteins (Fig 1B). Mammalian Apolipoprotein CI is a secreted molecule that contains a twenty-six amino acid N-terminal signal sequence that is co-translationally cleaved to yield a mature peptide [2]; similarly, the first twenty residues of clone 3–2 are predicted to encode a signal peptide. The gene encoding the 3–2 transcript maps to *Xenopus laevis* chromosome 7L (XLA7L) (*X*. *laevis* 9.2 on Jbrowse); there is no *apoCI*-like gene on XLA7S, suggesting that there is no second identifiable *apoCI*-like allogene in the *Xenopus laevis* genome. Taken together, our data suggest that 3–2 encodes the *Xenopus* homolog of ApoCI.

**Fig 2:**
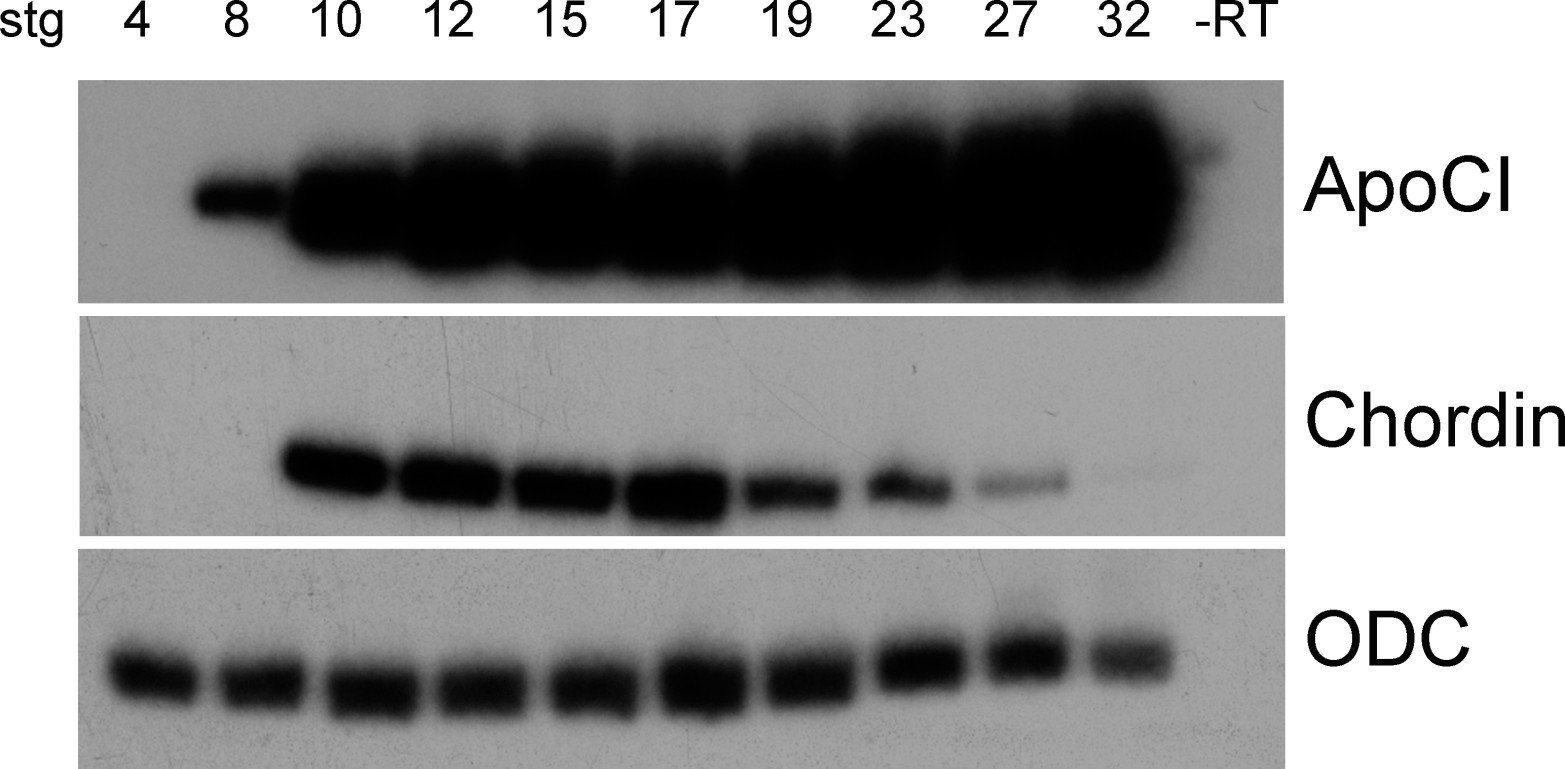
Temporal expression of a*poCI* mRNA. RT-PCR of RNA isolated from whole embryos between stage 4 and stage 32. *Chordin* serves as a staging control and *Ornithine decarboxylase* (ODC) as a control for input RNA levels.

The temporal expression of *Xenopus apoC1* was analyzed by reverse transcription polymerase chain reaction (RT-PCR) analysis of RNA extracted at various embryonic stages, from cleavage through early tadpole (Fig 2). *apoCI* expression is not observed at cleavage stage 4, suggesting that there is no maternal expression of this gene. *apoCI* expression is first detected at low levels at embryonic stage 8, which coincides with the midblastula transition, the start of zygotic gene expression [25]. Higher levels of *apoCI* expression are seen at stage 10, the onset of gastrulation, increasing progressively through stage 32.

**Fig 3:**
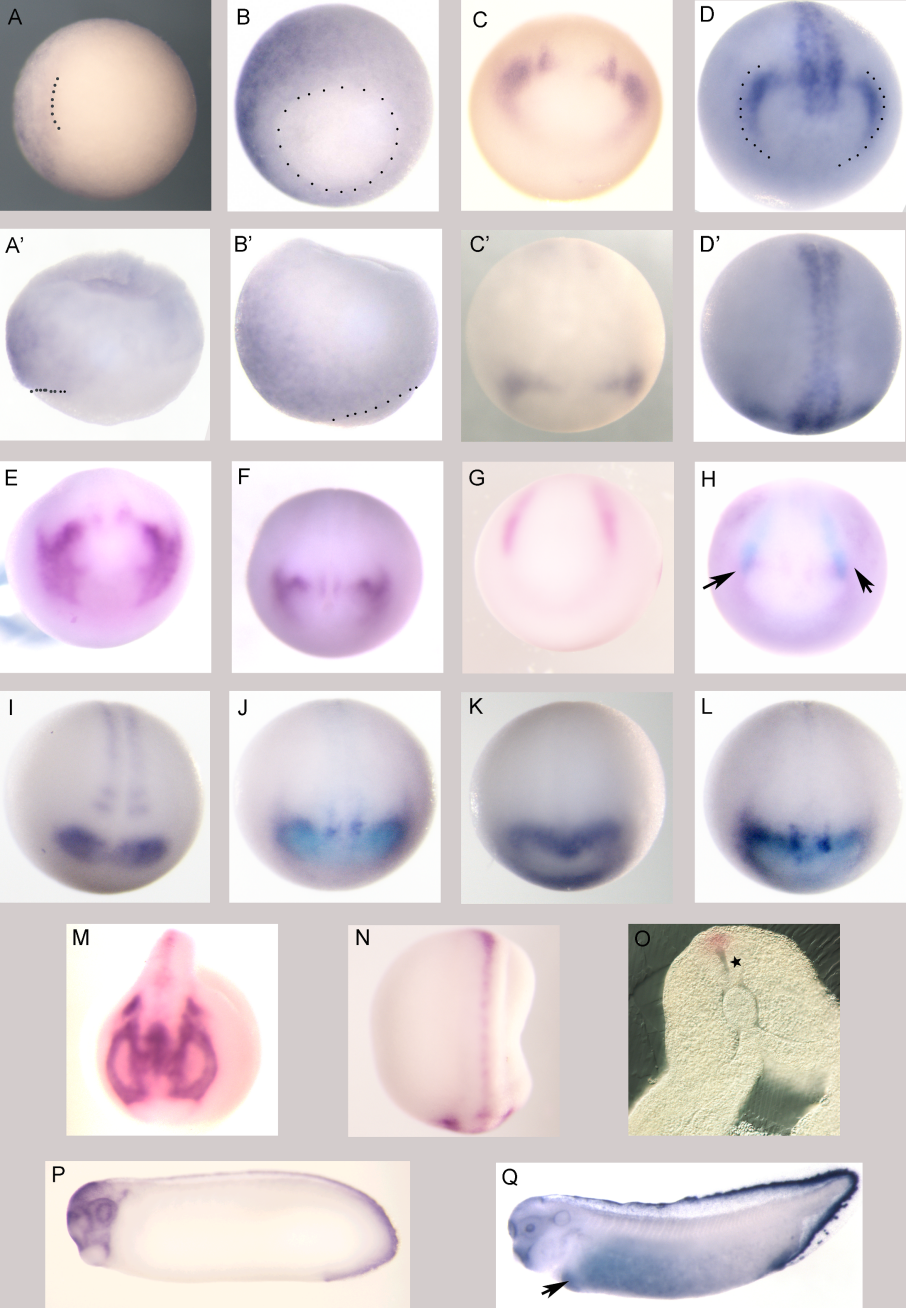
Spatial expression of *apoCI* mRNA. Vegetal (A, B) and lateral (A’, B’) views of stage 10 (A, A’) and stage 11 (B, B’) embryos, respectively; *a**poCI* transcript is localized to the dorsal ectoderm. Dotted lines in A, A’ mark the dorsal blastopore lip and dotted lines in B, B’ indicate the blastopore. Dorsal is to left in A, A’, B; dorsal is at 10 o’clock in B’. (C, D) Anterior views of *apoCI* expression in stage 13 and stage 15 embryos, respectively (dorsal to the top). Dotted line in D marks the pan-placodal primordium. (C’, D’) Dorsal views of embryos in C and D, respectively; anterior is down. (E) Anterior view of a stage 16 embryo; the anterior crescent of *apoCI* expression has expanded laterally. (F-L) Dorsoanterior views of stage 15 embryos; anterior is down and dorsal is to the top. (F) *apoCI* expression. (G) *slug* expression. (H) *apoCI* (purple) and *slug* (light blue) coexpression; arrows indicate region of overlap (cranial neural crest). (I) *pax6* expression. (J) *apoCI* (purple) and *pax6* (light blue) coexpression. (K) *otxA* expression. (L) *apoCI* (purple) and *otxA* (light blue) coexpression. (M, N) Anterior (M, dorsal is to the top) and dorsal (N, anterior is down) views showing *apoCI* expression in a stage 21 embryo. (O) Transverse section revealing expression of *apoCI* in the dorsal neural tube at stage 22; star indicates the neural tube. (P, Q) Lateral views of *apoCI* expression in stage 27 and stage 33 embryos, respectively; anterior is to the left. Arrow in Q marks liver primordium. No signal was detected with an *apoCI* sense strand probe (data not shown).

Whole mount *in situ* hybridization analysis with an antisense probe for *apoCI* revealed an interesting spatially-restricted expression pattern in *Xenopus* embryos beginning at early gastrula stages. *apoCI* expression becomes apparent at stage 10, when it is expressed in an area bordering the dorsal lip of the involuting marginal zone that extends animally to include the dorsal ectoderm, a region that contributes to the central nervous system later in development (Fig 3A and 3A’) [26, 27]. The dorsal localization of *apoCI* becomes more evident with increased expression levels as gastrulation progresses (Fig 3B and 3B’).

At stage 13, a*poCI* expression is seen in a crescent-like pattern in the anterior neural plate, in the region of the prospective lens and trigeminal placodes (Fig 3C and 3C’) [28]. As neurulation proceeds, expression also becomes visible in the dorsal midline (Fig 3D and 3D’). Anterior a*poCI* expression then expands laterally to include expression in the neural crest (Fig 3E). *In situ* hybridization using probes of both a*poCI* (Fig 3F) and the neural crest marker s*lug* (Fig 3G) reveals overlap of a*poCI* with the anteriormost region of *slug* expression marking the cranial neural crest (Fig 3H). Anterior expression of a*poCI* at neurula stage flanks the expression of lens marker p*ax6* (see Fig 3F, 3I and 3J) but shows overlap with the more posterior expression domain of forebrain marker o*txA* (see Fig 3F, 3K and 3L). After neural tube closure, expression of a*poCI* is seen in anterior neural tissue and in the cranial neural crest, including the region surrounding the developing eye; expression of *apoCI* is excluded from both the cement gland and the adenohypophyseal stomodeal anlage (Fig 3M)[29]. Transverse sections of early neurula embryos reveal that transient midline expression of a*poCI* is localized to the dorsal neural tube (Fig 3N and 3O). At the late tailbud stage, expression is visible surrounding the lens and in the olfactory placode, the trigeminal nerve, and the tailfin (Fig 3P). Diffuse *apoCI* expression is observed throughout the anterior endoderm at tadpole stages, with strong expression in the liver primordium (Fig 3Q). Dorsal and ventral tailfin expression increases markedly at tadpole stages: *apoCI* expression is observed at the distal margin of the fin, and at the border between the inner fin and outer fin where the latter structure is present; *apoCI* expression is also observed in scattered cells in both the dorsal and ventral outer fin (Fig 3Q and data not shown).

## Discussion

This work describes the identification and spatiotemporal expression of a*poCI* during early vertebrate embryogenesis. At gastrula stages, *Xenopus* a*poCI* expression is enriched in the dorsal ectoderm. During neurulation, a*poCI* is strongly expressed in the placodal primordium, the cranial neural crest and also the developing spinal cord. At tadpole stages expression is also seen in the anterior ventral endoderm, in the region of the presumptive liver, consistent with the strong expression of ApoCI in human and mouse liver.

The highly localized expression of *Xenopus* a*poCI* suggests a novel role for this factor in early neuroectodermal and/or cranial development, perhaps independent of its known role in lipid homeostasis. APOCI expression has been detected in human adult brain tissue, but analysis of localized expression is unavailable. In the Orange-spotted grouper, a*poCI* is also prominently expressed in the brain 7 days after hatching [30]. These studies suggest that ApoCI also functions during teleost and/or mammalian neurodevelopment.

Our studies do not directly address the mechanisms by which ApoCI may regulate steps in early development. The known functions of ApoCI in the transport of lipoproteins and modulation of binding to lipoprotein receptors raises the possibility that ApoCI may be involved in the modulation of lipid bound morphogens or lipoprotein receptors that are involved in early developmental signaling pathways, including those that mediate differentiation and/or patterning of the neurectoderm and neural border fates [4].

## Supporting information

S1 Fig Spatial expression of *apoCI* mRNA. Anterior view of stage 15 embryo; extended color reaction shown, for comparison with Fig 3F.
(TIFF)

## References

[1] Segrest JP, Jackson RL, Morrisett JD, Gotto AM Jr. A molecular theory of lipid-protein interactions in the plasma lipoproteins. FEBS Lett. 1974;38(3):247–58. Epub 1974/01/15. 0014-5793(74)80064-5 [pii]. . 4368333 10.1016/0014-5793(74)80064-5

[2] Knott TJ, Robertson ME, Priestley LM, Urdea M, Wallis S, Scott J. Characterisation of mRNAs encoding the precursor for human apolipoprotein CI. Nucleic Acids Res. 1984;12(9):3909–15. Epub 1984/05/11. ; PubMed Central PMCID: PMC318798. 6328444 10.1093/nar/12.9.3909 PMC318798

[3] Breslow JL. Apolipoprotein genetic variation and human disease. Physiol Rev. 1988;68(1):85–132. Epub 1988/01/01. doi: 10.1152/physrev.1988.68.1.85 . 2892216 10.1152/physrev.1988.68.1.85

[4] Weisgraber KH, Mahley RW, Kowal RC, Herz J, Goldstein JL, Brown MS. Apolipoprotein C-I modulates the interaction of apolipoprotein E with beta-migrating very low density lipoproteins (beta-VLDL) and inhibits binding of beta-VLDL to low density lipoprotein receptor-related protein. J Biol Chem. 1990;265(36):22453–9. Epub 1990/12/25. . 2266137

[5] Westerterp M, de Haan W, Berbee JF, Havekes LM, Rensen PC. Endogenous apoC-I increases hyperlipidemia in apoE-knockout mice by stimulating VLDL production and inhibiting LPL. J Lipid Res. 2006;47(6):1203–11. Epub 2006/03/16. M500434-JLR200 [pii] doi: 10.1194/jlr.M500434-JLR200 . 16537968 10.1194/jlr.M500434-JLR200

[6] Sehayek E, Eisenberg S. Mechanisms of inhibition by apolipoprotein C of apolipoprotein E-dependent cellular metabolism of human triglyceride-rich lipoproteins through the low density lipoprotein receptor pathway. J Biol Chem. 1991;266(27):18259–67. Epub 1991/09/25. . 1917954

[7] Jong MC, van Dijk KW, Dahlmans VE, Van der Boom H, Kobayashi K, Oka K, et al Reversal of hyperlipidaemia in apolipoprotein C1 transgenic mice by adenovirus-mediated gene delivery of the low-density-lipoprotein receptor, but not by the very-low-density-lipoprotein receptor. Biochem J. 1999;338 (Pt 2):281–7. Epub 1999/02/20. ; PubMed Central PMCID: PMC1220053. 10024503 PMC1220053

[8] Berbee JFP, van der Hoogt CC, Sundararaman D, Havekes LM, Rensen PCN. Severe hypertriglyceridemia in human APOC1 transgenic mice is caused by apoC-I-induced inhibition of LPL. J Lipid Res. 2005;46(2):297–306. doi: 10.1194/jlr.M400301-JLR200 15576844 10.1194/jlr.M400301-JLR200

[9] Westerterp M, Van Eck M, de Haan W, Offerman EH, Van Berkel TJC, Havekes LM, et al Apolipoprotein CI aggravates atherosclerosis development in ApoE-knockout mice despite mediating cholesterol efflux from macrophages. Atherosclerosis. 2007;195(1):e9–e16. doi: 10.1016/j.atherosclerosis.2007.01.015 17320883 10.1016/j.atherosclerosis.2007.01.015

[10] Hamsten A, Silveira A, Boquist S, Tang R, Bond MG, de Faire U, et al The apolipoprotein CI content of triglyceride-rich lipoproteins independently predicts early atherosclerosis in healthy middle-aged men. J Am Coll Cardiol. 2005;45(7):1013–7. Epub 2005/04/06. S0735-1097(04)02579-3 [pii] doi: 10.1016/j.jacc.2004.12.049 . 15808756 10.1016/j.jacc.2004.12.049

[11] Lauer SJ, Walker D, Elshourbagy NA, Reardon CA, Levy-Wilson B, Taylor JM. Two copies of the human apolipoprotein C-I gene are linked closely to the apolipoprotein E gene. J Biol Chem. 1988;263(15):7277–86. Epub 1988/05/25. . 2835369

[12] Poduslo SE, Neal M, Herring K, Shelly J. The apolipoprotein CI A allele as a risk factor for Alzheimer's disease. Neurochem Res. 1998;23(3):361–7. Epub 1998/03/03. . 9482248 10.1023/a:1022409617539

[13] Ki CS, Na DL, Kim DK, Kim HJ, Kim JW. Genetic association of an apolipoprotein C-I (APOC1) gene polymorphism with late-onset Alzheimer's disease. Neurosci Lett. 2002;319(2):75–8. Epub 2002/02/05. S0304394001025599 [pii]. . 11825674 10.1016/s0304-3940(01)02559-9

[14] Xu Y, Berglund L, Ramakrishnan R, Mayeux R, Ngai C, Holleran S, et al A common Hpa I RFLP of apolipoprotein C-I increases gene transcription and exhibits an ethnically distinct pattern of linkage disequilibrium with the alleles of apolipoprotein E. J Lipid Res. 1999;40(1):50–8. Epub 1998/12/31. . 9869649

[15] Strittmatter WJ, Saunders AM, Schmechel D, Pericak-Vance M, Enghild J, Salvesen GS, et al Apolipoprotein E: high-avidity binding to beta-amyloid and increased frequency of type 4 allele in late-onset familial Alzheimer disease. Proc Natl Acad Sci U S A. 1993;90(5):1977–81. Epub 1993/03/01. ; PubMed Central PMCID: PMC46003. 8446617 10.1073/pnas.90.5.1977 PMC46003

[16] Abildayeva K, Berbee JFP, Blokland A, Jansen PJ, Hoek FJ, Meijer O, et al Human apolipoprotein C-I expression in mice impairs learning and memory functions. J Lipid Res. 2008;49(4):856–69. doi: 10.1194/jlr.M700518-JLR200 18160739 10.1194/jlr.M700518-JLR200

[17] Dulac C, Axel R. A novel family of genes encoding putative pheromone receptors in mammals. Cell. 1995;83(2):195–206. Epub 1995/10/20. 0092-8674(95)90161-2 [pii]. . 7585937 10.1016/0092-8674(95)90161-2

[18] Hemmati-Brivanlou A, de la Torre JR, Holt C, Harland RM. Cephalic expression and molecular characterization of Xenopus En-2. Development. 1991;111(3):715–24. Epub 1991/03/01. . 1679005 10.1242/dev.111.3.715

[19] Nieuwkoop P, Faber J. ‘Normal table of Xenopus Laevis’. North Holland Publishing Co, Amsterdam, The Netherlands 1967.

[20] Wilson PA, Hemmati-Brivanlou A. Induction of epidermis and inhibition of neural fate by Bmp-4. Nature. 1995;376(6538):331–3. Epub 1995/07/27. doi: 10.1038/376331a0 . 7630398 10.1038/376331a0

[21] Harland RM. In situ hybridization: an improved whole-mount method for Xenopus embryos. Methods Cell Biol. 1991;36:685–95. Epub 1991/01/01. . 1811161 10.1016/s0091-679x(08)60307-6

[22] Koga M, Kudoh T, Hamada Y, Watanabe M, Kageura H. A new triple staining method for double in situ hybridization in combination with cell lineage tracing in whole-mount Xenopus embryos. Dev Growth Differ. 2007;49(8):635–45. Epub 2007/08/23. DGD958 [pii] doi: 10.1111/j.1440-169X.2007.00958.x . 17711476 10.1111/j.1440-169X.2007.00958.x

[23] Linker C, Bronner-Fraser M, Mayor R. Relationship between Gene Expression Domains of Xsnail, Xslug, and Xtwist and Cell Movement in the Prospective Neural Crest of Xenopus. Developmental Biology. 2000;224(2):215–25. doi: 10.1006/dbio.2000.9723 10926761 10.1006/dbio.2000.9723

[24] Hirsch N, Harris WA. *Xenopus* Pax-6 and retinal development. Journal of Neurobiology. 1997;32(1):45–61. 8989662

[25] Newport J, Kirschner M. A major developmental transition in early Xenopus embryos: I. characterization and timing of cellular changes at the midblastula stage. Cell. 1982;30(3):675–86. Epub 1982/10/01. 0092-8674(82)90272-0 [pii]. . 6183003 10.1016/0092-8674(82)90272-0

[26] Gould SE, Grainger RM. Neural induction and antero-posterior patterning in the amphibian embryo: past, present and future. Cell Mol Life Sci. 1997;53(4):319–38. Epub 1997/04/01. . 9137624 10.1007/PL00000609 PMC11147268

[27] Slack JM, Isaacs HV, Johnson GE, Lettice LA, Tannahill D, Thompson J. Specification of the body plan during Xenopus gastrulation: dorsoventral and anteroposterior patterning of the mesoderm. Dev Suppl. 1992:143–9. Epub 1992/01/01. . 1299359

[28] Schlosser G, Ahrens K. Molecular anatomy of placode development in Xenopus laevis. Dev Biol. 2004;271(2):439–66. Epub 2004/06/30. doi: 10.1016/j.ydbio.2004.04.013 [pii]. . 15223346 10.1016/j.ydbio.2004.04.013

[29] Dickinson A, Sive H. Positioning the extreme anterior in Xenopus: cement gland, primary mouth and anterior pituitary. Seminars in cell & developmental biology. 2007;18(4):525–33. doi: 10.1016/j.semcdb.2007.04.002 . 17509913 10.1016/j.semcdb.2007.04.002

[30] Wang Y, Zhou L, Li Z, Gui JF. Molecular cloning and expression characterization of ApoC-I in the orange-spotted grouper. Fish Physiol Biochem. 2008;34(4):339–48. Epub 2008/10/30. doi: 10.1007/s10695-007-9193-y . 18958591 10.1007/s10695-007-9193-y

