# Supplementary figures and images for: Cloning and spatiotemporal expression of Xenopus laevis Apolipoprotein CI
Source: PLoS One. 2018 Jan 18;13(1):e0191470. doi: 10.1371/journal.pone.0191470 (PMC5773212; doi:10.1371/journal.pone.0191470)

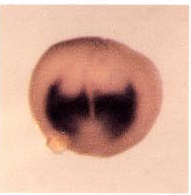

Supplement: S1 Fig — Anterior view of stage 15 embryo; extended color reaction shown, for comparison with Fig 3F. (TIFF) [file pone.0191470.s001.tiff]
